# Sock-Type Wearable Sensor for Estimating Lower Leg Muscle Activity Using Distal EMG Signals

**DOI:** 10.3390/s19081954

**Published:** 2019-04-25

**Authors:** Takashi Isezaki, Hideki Kadone, Arinobu Niijima, Ryosuke Aoki, Tomoki Watanabe, Toshitaka Kimura, Kenji Suzuki

**Affiliations:** 1 NTT Service Evolution Laboratories, Nippon Telegraph and Telephone Corporation, 1-1 Hikarinooka, Yokosuka, Kanagawa 239-0847, Japan; ryousuke.aoki.nz@hco.ntt.co.jp (R.A.); tomoki.watanabe.cd@hco.ntt.co.jp (T.W.); 2 Center for Cybernics Research, University of Tsukuba, 1-1-1 Tennodai, Tsukuba, Ibaraki 305-8573, Japan; kadone@ai.iit.tsukuba.ac.jp; 3 NTT Plala Inc., TOC Ariake East Tower 14F, 3-5-7 Ariake, Koto-ku, Tokyo 135-0063, Japan; niijima@plala.co.jp; 4 NTT Communication Science Laboratories, 3-1 Morinosato-Wakamiya, Atsugi, Kanagawa 243-0198, Japan; toshitaka.kimura.kd@hco.ntt.co.jp; 5 Faculty of Engineering, Information and Systems, University of Tsukuba, 1-1-1 Tennodai, Tsukuba, Ibaraki 305-8573, Japan; kenji@ieee.org

**Keywords:** wearable, distal EMG signal, muscle activity estimation

## Abstract

Lower leg muscle activity contributes to body control; thus, monitoring lower leg muscle activity is beneficial to understand the body condition and prevent accidents such as falls. Amplitude features such as the mean absolute values of electromyography (EMG) are used widely for monitoring muscle activity. Garment-type EMG measurement systems use electrodes and they enable us to monitor muscle activity in daily life without any specific knowledge and the installation for electrode placement. However, garment-type measurement systems require a high compression area around the electrodes to prevent electrode displacement. This makes it difficult for users to wear such measurement systems. A less restraining wearable system, wherein the electrodes are placed around the ankle, is realized for target muscles widely distributed around the shank. The signals obtained from around the ankle are propagated biosignals from several muscles, and are referred to as *distal EMG signals*. Our objective is to develop a sock-type wearable sensor for estimating lower leg muscle activity using distal EMG signals. We propose a signal processing method based on multiple bandpass filters from the perspectives of noise separation and feature augmentation. We conducted an experiment for designing the hardware configuration, and three other experiments for evaluating the estimation accuracy and dependability of muscle activity analysis. Compared to the baseline based on a 20-500 Hz bandpass filter, the results indicated that the proposed system estimates muscle activity with higher accuracy. Experimental results suggest that lower leg muscle activity can be estimated using distal EMG signals.

## 1. Introduction

Lower leg muscle activity is important for controlling the body. Relationships between lower leg muscle activity and the risk of falls among the elderly have already been reported [1,2]; delayed plantar flexion increases the risk of such falls. In addition, there is a certain amount of people in the young group (20–45 years) that experience falling [3]. The ability to recover balance after a fall is related to lower leg muscle activity [4]; further the time and volume of muscle activities are important for recovering balance. The relationship between the lower leg muscle activity and certain anomalies such as the freezing of gait in Parkinson’s disease, has also been studied [5]. Training the lower leg muscles contributes to improvements in terms of gait, such as velocity, cadence, step time and so on [6]. Thus, monitoring lower leg muscle activity when walking is beneficial for understanding the body conditions and preventing accidents. Currently, muscle activity analysis is conducted in specific facilities, such as hospitals and universities owing to the requirement of specific knowledge of kinematics and anatomy.

Electromyography (EMG) is used widely for monitoring muscle activity. Biopotential signals that activating muscle fibers propagate from the neuromuscular junction to the tendon along the muscle fibers. An electrical field is created by the stimulation from the neuron activating the muscle fiber’s chemical receptors [7]. By placing electrodes on the surface of the skin, biopotential signals are measured as EMG signals. An EMG signal-based system has several applications [8,9,10]. The simplification of EMG signal measurement is considered important for further popularization; however, it is necessary to select the muscle site to be measured, and to determine the position where the electrode is to be placed, based on kinematic and anatomical knowledge. It is difficult for users who have no kinematic and anatomical knowledge to place electrodes on specific muscles. With garment-type measurement systems, electrodes placements are included in the action of wearing socks. Therefore, garment-type measurement systems can be applied for daily use. There have been studies on conductive fabric-type EMG measurement materials for garment-type EMG measurement systems [11,12]. Myers et al. presented a silver nanowire (AgNW)-based dry electrode for electrophysiological wearable monitoring [12]. They compared EMG signals measured using AgNW dry electrodes and conventional Ag/AgCl wet electrodes, and they confirmed that there were a few differences. Nippon Telegraph and Telephone Corporation and Toray developed “hitoe^®^”, which is made of electro-conductive polymer nanofibers [11]. Their technology can be applied to users who have metal allergies because it is not made of metal. Some other types of conductive fabric have also become commercially available [13].

To increase the opportunities for monitoring lower leg muscle activity, we focus on garment-type measurement systems using conductive fabric. Users do not need to place electrodes themselves, because conductive fabric electrodes are already implemented in garment-type measurement systems. However, garment-type measurement systems need high compression around the electrodes for preventing electrode displacement. If electrodes are distributed on a large area in a system, the area that requires high compression also expands, which makes it difficult for users to wear the electrodes. Considering the applications for daily use among a wide variety of users, including the elderly, it is important that the measurement system be easy to wear.

Accurate and repeatable EMG signals can be used for the analysis of signal amplitude, spectral variables and muscle fiber conduction velocity. Rainoldi et al. explored optimal electrode positions for obtaining accurate and repeatable EMG signals [14]. It is common to measure EMG signals by placing a sensor electrode at the center of each target muscle. Such signals propagate to and from neighboring muscles [15]. Therefore, it should be possible to measure EMG signals by placing electrodes at the distal positions close to the tendons of target muscles. We define biosignals not measured on the muscle bellies as *distal EMG signal*. Using distal EMG signals, electrode positions can be designated for each situation based on propagation characteristics. Some studies used distal EMG signals to estimate facial expressions [16,17]. They revealed that distal EMG signals can be used for discriminating between smiles, frowns, and neutral facial expressions. Independent component analysis and artificial neural networks were used for separating signals and recognizing facial expressions, respectively. However, studies on the distal EMG-based analysis of other muscles, such as the lower leg, are lacking.

**Figure 1:**
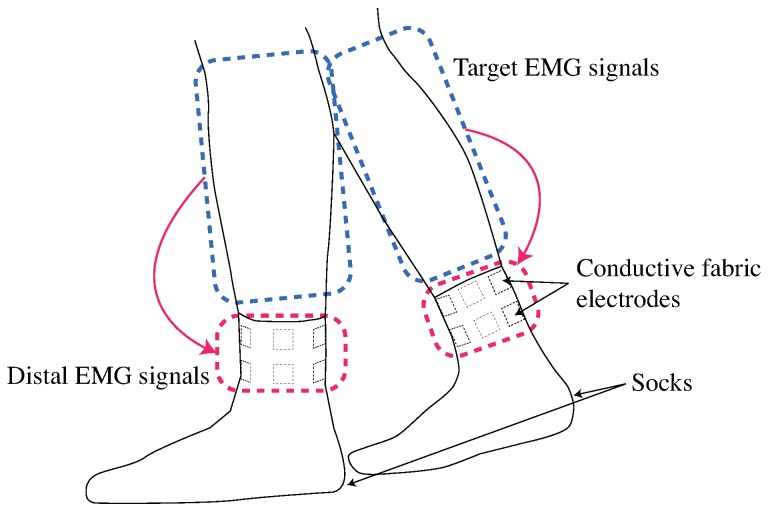
Design of socks that measure distal EMG signals propagated target muscles.

Our objective is to develop a sock-type wearable sensor consisting of electrodes that are implemented around the ankle for estimating lower leg muscle activity. Figure 1 shows the concept for the proposed system. The proposed system requires high compression in the red area because electrodes are placed around the ankle, while high compression is required in the blue area if electrodes are positioned on each muscle belly. From a wearing pressure point of view, the proposed system is minimally restraining and easy to wear. The signals obtained from around the ankle are the distal EMG signals; which are the propagated biopotential signals of target muscles. The time and volume of muscle activity is a major target of muscle activity analysis [18,19]. We construct the EMG signal estimation system based on the distal EMG signals for the temporal-spatial muscle activity analysis.

Amplitude and spectral variables are the main features obtained from EMG signals [20]. Amplitude features contain information on muscle force ratio, muscle activation (ON/OFF) state, and the timing of muscle contraction and relaxation. There are several gait analysis applications based on the amplitude features of EMG signals [2,21]. It was reported that the mean absolute values represent amplitude features more than other amplitude feature calculations [22]. Therefore, mean absolute values are adopted as a muscle activity in this study.

The main contributions of this paper are:

- To verify that distal EMG signals can be used for estimating lower leg muscle activity
- To design low restrained electrodes positions for ease of wearing
- To implement the signal processing of mean absolute values estimation based on the distal EMG signals

We outline our system’s configuration in Section 2. Three analyses from the data of one experiment for evaluating the accuracy for estimating EMG signals based on distal EMG signals are described in Section 3. We explain the experimental results in Section 4, and describe the discussion for results in Section 5. The conclusion is described in Section 6.

## 2. System Configuration

Our proposed system estimates amplitude features by using distal EMG signals, while the conventional system measures them directly. There are six target muscles that are focused on in many studies to monitor lower leg muscle activity: *tibialis anterior*, *gastrocnemius lateral*, *gastrocnemius medial*, *soleus lateral*, *soleus medial*, *and peroneus muscle* [1,2,4,23]. This section describes the construction of the sock for measuring distal EMG signals and the estimation algorithm.

### 2.1. Construction of Distal EMG Signal Measurement Sock

Socks are an appropriate medium to implement our EMG measurement system for daily measurement as people usually wear socks every day for many types of activities, and therefore, resistance to wearing socks is low. Moreover, socks, especially around the ankle, are tight and do not slide down. This makes them effective for measuring signals because they allow electrodes to remain in contact with the skin without significant displacement.

#### 2.1.1. Electrodes Position Design

**Figure 2:**
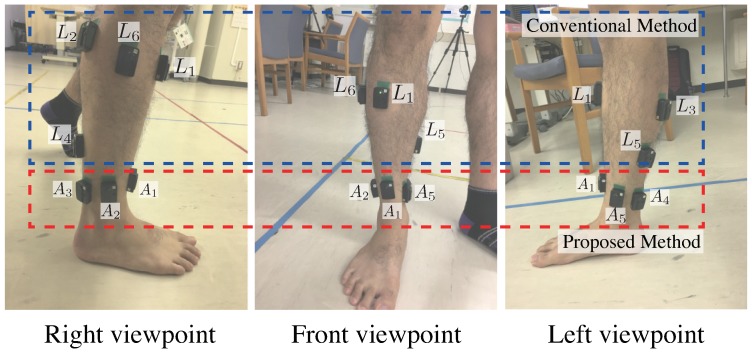
Sensors positions for the right lower leg. $A_{1}$-$A_{5}$ are the proposed positions. EMG signals from targeted muscles are obtained from $L_{1}$-$L_{6}$.

To develop our system for estimating lower leg muscle activity based on distal EMG signals, the position and number of electrodes are important. Previously, we explored the placement of electrodes using the Trigno EMG system (Delsys, Inc., Natick, MA, USA) [24]. We conducted an experiment using four male participants before developing the socks. As shown in Figure 2, sensors were placed on the target muscles of the lower right leg. The relationships between the sensor labels and the muscles are summarized in Table 1. Position $A_{1}$ was located on the tendon of the tibialis anterior, $A_{3}$ and $A_{4}$ were arranged so that the Achilles tendon would be between them, $A_{2}$ was located between $A_{1}$ and $A_{3}$, and $A_{5}$ was located between $A_{4}$ and $A_{1}$. Each subject conducted plantar flexion and dorsiflexion for 30 s, and biopotential signals were measured. This experiment described in this section was conducted with approval from the ethics committee of the University of Tsukuba (2017R157) and NTT Service Evolution Laboratories (ERP-Ethics-17-013).

A notch filter was applied to all signals for reducing the power-supply noise. It has been reported that the bandwidth of usable energy for surface EMG signals is between 20–500 Hz [25]. Further, this bandwidth is used in clinical research [26]. The sampling rate is in accordance with the Nyquist theorem, which states that the sampling rate must be greater than twice the highest frequency component of the analog signal. The sampling rate of the sensors was set to 2 kHz which is the default setting of the Trigno EMG system. A frequency of 2 kHz is sufficient from the Nyquist theorem point of view. A high-pass filter with a cut-off frequency of 20 Hz is recommended for general noise reduction [27]. All signals were filtered with a bandpass filter with passing frequencies from 20 to 500 Hz. The mean absolute values were calculated with the following equations.

(1) $$S_{L_{i}}\left(k\right)=\frac{1}{\tau }\sum_{j=0}^{\tau -1}\left(\right|l_{i}\left(k-j\right)\left|\right)$$

(2) $$S_{A_{i}}\left(k\right)=\frac{1}{\tau }\sum_{j=0}^{\tau -1}\left(\right|a_{i}\left(k-j\right)\left|\right)$$

(3) $$L\left(k\right)={\left[S_{L_{1}}\left(k\right),\;S_{L_{2}}\left(k\right),\;S_{L_{3}}\left(k\right),\;S_{L_{4}}\left(k\right),\;S_{L_{5}}\left(k\right),\;S_{L_{6}}\left(k\right)\right]}^{T}$$

(4) $$A\left(k\right)={\left[S_{A_{1}}\left(k\right),\;S_{A_{2}}\left(k\right),\;S_{A_{3}}\left(k\right),\;S_{A_{4}}\left(k\right),\;S_{A_{5}}\left(k\right)\right]}^{T}$$

where *i* is the number of the position label in Figure 2, $l_{i}$ and $a_{i}$ are the vectors of samples measured from $L_{i}$ and $A_{i}$, $l_{i}\left(k\right)$ and $a_{i}\left(k\right)$ are the *k*-th samples of $l_{i}$ and $a_{i}$; $S_{L_{i}}\left(k\right)$ is the *k*-th mean absolute value of $l_{i}$, $S_{A_{i}}\left(k\right)$ is the *k*-th mean absolute value of $a_{i}$. L(k) and A(k) are the vectors of mean absolute values of $S_{L_{1}}\left(k\right),\;$ $S_{L_{2}}\left(k\right),\;$ $S_{L_{3}}\left(k\right),\;$ $S_{L_{4}}\left(k\right),\;$ $S_{L_{5}}\left(k\right),\;$ and $S_{L_{6}}\left(k\right)$ and $S_{A_{1}}\left(k\right),\;S_{A_{2}}\left(k\right),\;S_{A_{3}}\left(k\right),\;S_{A_{4}}\left(k\right),\;$ and $\;S_{A_{5}}\left(k\right)$. τ is the width of the mean absolute value. In this paper, the time width of the mean absolute value calculation was set to 0.1 s. Then, τ was set to 200 based on the sampling rate and the time width of the mean absolute value. We calculated a linear regression model, which is a basic machine learning approach that satisfies the following equation [28].

(5) L(k)=MA(k)+b

where ***M***, which is composed of 6 × 5 dimensions, is the array of the weights of each explanatory variable for the objective variable; ***b*** is a bias; and ***L*** and ***A*** contain all samples of L(k) and A(k), respectively. Regression signals $\hat{L}$ are calculated using ***M***, ***A***, and ***b***. We used the correlation coefficient between the measured mean absolute values ***L*** and the estimated mean absolute values $\hat{L}$ as a metric for comparing estimation accuracy.

Each ***L*** and ***A*** of each subject were split into two: training data and test data. Splitting data for obtaining the training and test data is a general machine learning approach [29]. Each ***L*** and ***A*** of each subject comprises 60,000 samples (2 kHz × 30 s). The first half of every data point consists of 1–30,000-th samples, and it is used as the training data. The second half consists 30,001–60,000-th samples, and it is used as test data. In this experiment, four training data and four test data were extracted from four subjects. Training data and test data were concatenated individually. Concatenated training data were used for model training and the concatenated test was used for regression. Estimated data that contain 6 channels signals of target muscle was calculated. In consideration of training data dependency, same estimation was performed by replacing the training data and the test data. $\hat{L}$ data were obtained by concatenating two estimated data. Correlation coefficients were calculated using ***L*** and $\hat{L}$.

**Figure 3:**
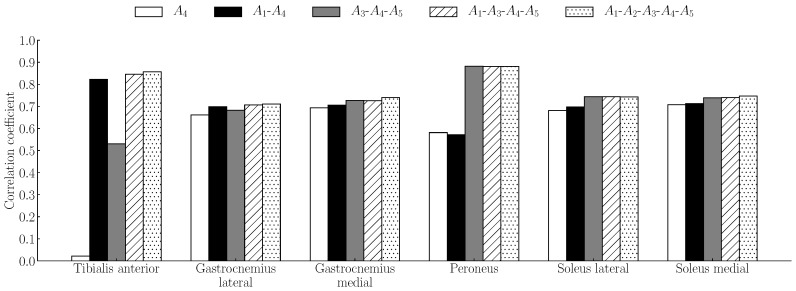
Correlation coefficients between the measured signals and the estimated signals. Five combinations whose median value of correlation coefficients was the highest in each number of sensors. The correlation coefficients of [$A_{1}$-$A_{2}$-$A_{3}$-$A_{4}$-$A_{5}$] combination was the highest from those of other combinations in all muscles.

In order to determine the number and position of sensors, all combinations of the number and positions of sensors were tested. At each number of sensors, the sensor combination was calculated. In the case of one sensor, the number of sensor combinations was five: [$A_{1}$], [$A_{2}$], [$A_{3}$], [$A_{4}$], and [$A_{5}$]. In the case of two sensors, the number of sensor combinations was 10: [$A_{1}$-$A_{2}$], [$A_{1}$-$A_{3}$], [$A_{1}$-$A_{4}$], [$A_{1}$-$A_{5}$], [$A_{2}$-$A_{3}$], [$A_{2}$-$A_{4}$], [$A_{2}$-$A_{5}$], [$A_{3}$-$A_{4}$], [$A_{3}$-$A_{5}$], and [$A_{4}$-$A_{5}$]. The number of sensors was up to five. Six correlation coefficients for six target muscles were calculated from one combination. We determined the combination whose correlation coefficients are the highest as the appropriate combination. All *p*-values were less than 0.05. Figure 3 shows the correlation coefficients of five combinations whose median value of correlation coefficients was the highest in each number of sensors. The correlation coefficients of [$A_{1}$-$A_{2}$-$A_{3}$-$A_{4}$-$A_{5}$] combination was the highest from those of other combinations in all muscles. Our results indicated that five channels around the ankle are appropriate for measuring distal EMG signals and estimating the activity of the lower leg muscles.

#### 2.1.2. Materials

**Figure 4:**
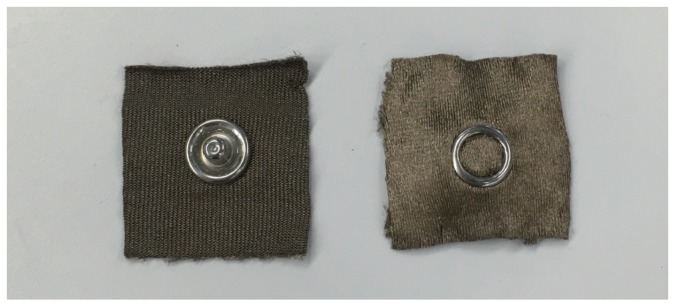
Conductive fabric electrodes where electrode surface contacts skin (**right**) and electrode surface contacts socks (**left**). Electric signals are measured through snaps located at center of electrodes.

To implement our measurement system into socks, electrodes should be made of fabric. In this study, we used a conductive knit fabric made of silver-plated nylon available from SparkFun [13]. Figure 4 shows an example of a conductive fabric electrode. Electric signals are measured through snaps located at the center of the electrodes. The conductive fabric and snaps are used to obtain biopotential signals. The snaps are made of brass.

**Figure 5:**
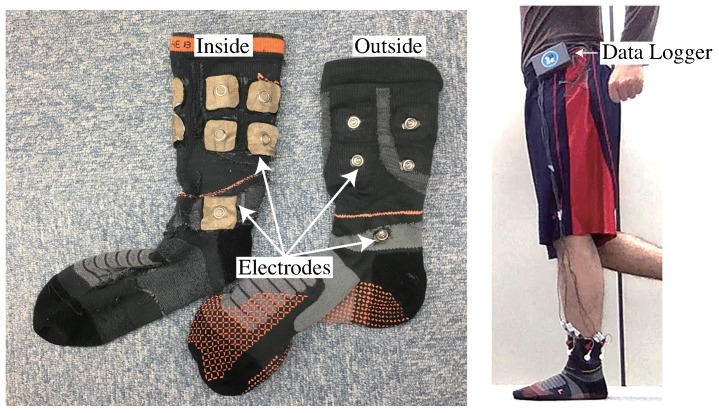
(**Left**): configuration of sock-type wearable sensor for estimating lower leg muscle activity with 10 conductive-fabric electrodes around the ankle to measure five channels; a conductive-fabric electrode placed on medial malleolus, acting as the ground. (**Right**): patient wearing socks; each side of the sock estimates activity of each lower leg muscle and each snap is connected to the data logger via wires.

The left side in Figure 5 shows the configuration of a sock-type wearable sensor for estimating lower leg muscle activity. The electrode surface on the inner surface of the sock contacts the skin. Ten conductive fabric electrodes are placed around the ankle to measure five channels. A conductive fabric electrode is placed on the medial malleolus for a ground signal. There are 11 snaps, and signals are obtained through these snaps from the outer surface. The right side shows an image of a person wearing the socks. Each snap is connected to the data logger via wires. The data logger accumulates all signals, the signal data are extracted from the logger after measurements, and they are analyzed. Each side of the sock estimates the activity of each side of lower leg muscles.

**Figure 6:**
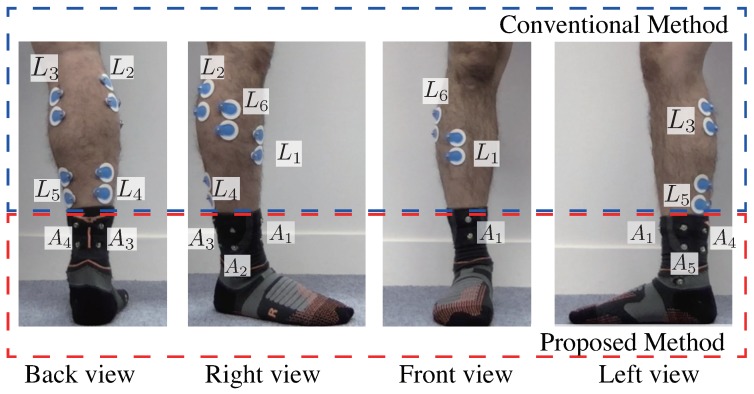
Electrode positions for the right lower leg. $A_{1}$-$A_{5}$ are proposed positions. EMG signals from targeted muscles are obtained from $L_{1}$-$L_{6}$.

Position $A_{1}$ was located on the tendon of the tibialis anterior, $A_{3}$ and $A_{4}$ were arranged so that the Achilles tendon would be between them, $A_{2}$ was located between $A_{1}$ and $A_{3}$, and $A_{5}$ was located between $A_{4}$ and $A_{1}$. Wet type electrodes were placed on $L_{1}$-$L_{6}$, as shown in Figure 6.

### 2.2. Activity Estimation

**Figure 7:**
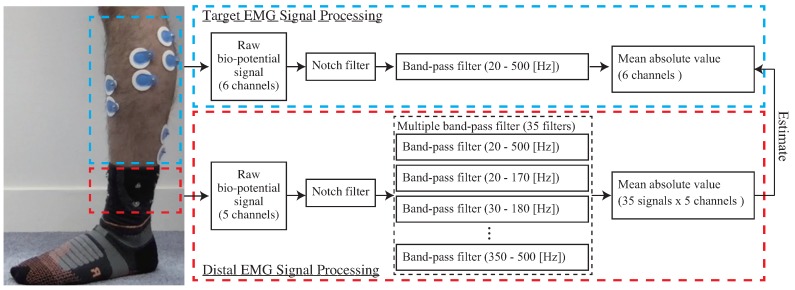
Diagram of EMG processing.

Distal EMG signals contain the EMG signal of the target muscle, crosstalk derived from EMG signals of other muscles, and artifacts derived from movements. Extracting effective features is important to estimate target EMG signal at high accuracy; therefore, signal processing for noise separation and various features extraction is needed. We adopted the multiple band-pass filter for signal processing. Figure 7 shows the EMG processing. The proposed system involves calibration and estimation phases to estimate the target muscle activity from the distal EMG signals.

#### 2.2.1. Calibration

The goal of this phase is to construct the estimation model. First, target EMG signals ($l_{1}$, $l_{2}$, $l_{3}$, $l_{4}$, $l_{5}$, and $l_{6}$) and distal EMG signals ($a_{1}$, $a_{2}$, $a_{3}$, $a_{4}$, and $a_{5}$) for specific tasks are measured in parallel. The $l_{i}$ is measured from $L_{i}$, as shown in Table 1. The distal EMG signals are measured using the conductive fabric electrodes placed in the socks, and the EMG signals of target muscles are measured using wet-type electrodes. Choosing calibration tasks depends on target tasks because the activity of the target muscles, crosstalk, and artifacts of the calibration tasks need to cover those of the target tasks. For removing the power-supply noise, the notch filter was applied. For removing general motion artifacts, target EMG signals ($l_{1}$, $l_{2}$, $l_{3}$, $l_{4}$, $l_{5}$, $l_{6}$) are filtered with a bandpass filter with passing frequencies from 20–500 Hz. As for noise separation and feature augmentation, we propose a feature extraction method based on multi-bandpass filters. The array of $A_{i}$ from the following equation. The first component in the array is the signal filtered with a 20–500 Hz bandpass filter to remove motion artifacts. The following components are the signal filtered with a 150 [Hz] window bandpass filter shifted 10 [Hz] at a time. These parameters were experimentally determined. We then obtain the $A_{i}$ array from the following equation.

(6) $$S_{\left(A_{i},\;20-500\;\mathrm{Hz}\right)}\left(k\right)=\frac{1}{\tau }\sum_{j=0}^{\tau -1}\{|BPF\left(a_{i},20-500\;\mathrm{Hz}\right)\left(k-j\right)\left|\right\}$$

(7) $$\begin{array}{cc} A_{i}\left(k\right)=[ & S_{\left(A_{i},\;20-500\;\mathrm{Hz}\right)}\left(k\right),\;S_{\left(A_{i},\;20-170\;\mathrm{Hz}\right)}\left(k\right),\;S_{\left(A_{i},\;30-180\;\mathrm{Hz}\right)}\left(k\right),\;\\ & S_{\left(A_{i},\;40-190\;\mathrm{Hz}\right)}\left(k\right),\;\ldots ,\;S_{\left(A_{i},\;340-490\;\mathrm{Hz}\right)}\left(k\right),\;S_{\left(A_{i},\;350-500\;\mathrm{Hz}\right)}\left(k\right)] \end{array}$$

where $a_{i}$ is the distal EMG signal measured from $A_{i}$, *i* is the position label, and $BPF(a_{i},p-q\;\mathrm{Hz})$ is the signal filtered with a *p*-*q* [Hz] bandpass filter of $a_{i}$. Eventually, 35 signals are calculated from each of the five channels around the ankle. $L\left(k\right)={\left[S_{L_{1}}\left(k\right),\;S_{L_{2}}\left(k\right),\;S_{L_{3}}\left(k\right),\;S_{L_{4}}\left(k\right),\;S_{L_{5}}\left(k\right),\;S_{L_{6}}\left(k\right)\right]}^{T}$ is calculated as Equation (3). A(k) is calculated as follows.

(8) $$A\left(k\right)=[A_{1}\left(k\right),\;A_{2}\left(k\right),\;A_{3}\left(k\right),\;A_{4}\left(k\right),\;A_{5}\left(k\right)]$$

As mentioned, distal EMG signals contain not only target EMG signals but also crosstalk and artifacts. These crosstalk and artifacts mix into the distal EMG signals nonlinearly. We adopted a nonlinear regression model and chose a gradient boosting regressor as a nonlinear estimating approach. The key property of linear regression models is that it is a linear function of the parameters to input values [28]. Nonlinear models are those that cannot be described by linear models. The gradient tree boosting algorithm follows an approach wherein new regression trees are added for reducing the residual error against the true values [30]. The gradient tree boosting algorithm is described as Algorithm 1. Algorithm 1 shows the case to construct the estimation model $M_{L_{i}}$ against $L_{i}$.
**Algorithm 1** Gradient Tree Boosting Algorithm.Initialize $M_{\left(0,L_{i}\right)}\left(x\right)$ = arg min${}_{\gamma }\sum_{k=1}^{N}Q\left(S_{L_{i}}\left(k\right),\gamma \right)$For *h* = 1 to *H*:(a)For *k* = 1,2,…,N compute
$$r_{kh}=-{\left[\frac{\partial Q(S_{L_{i}}\left(k\right),M\left(A\left(k\right)\right))}{\partial M(A(k))}\right]}_{M=M_{\left(h-1,L_{i}\right)}}$$(b)Fit a regression tree to the targets $r_{kh}$ giving terminal regions $R_{vh},v=1,2,\ldots ,V_{h}$(c)For $v=1,2,\ldots ,V_{h}$ compute
$$\gamma_{vh}={\arg\min}_{\gamma }\sum_{A\left(k\right)\in R_{vh}}Q\left(S_{L_{i}}\left(k\right),M_{\left(h-1,L_{i}\right)}\left(A\left(k\right)\right)+\gamma \right)$$(d)Update $M_{\left(h,L_{i}\right)}\left(x\right)=M_{\left(h-1,L_{i}\right)}\left(x\right)+\sum_{v=1}^{V_{h}}\gamma_{vh}I\left(x\in R_{vh}\right)$Finalize $M_{\left(H,L_{i}\right)}$ as $M_{L_{i}}$
where *x* is any one sample from the array A = A(k),k=(1,…,N), $V_{m}$ is the division number of regions at the *m*-th iteration, *I* is an indicator function, and *Q* is a loss function.

Biopotential signals, which are measured from the surface of the skin, vary depending on muscle masses, skin impedance, and coordination of muscle activity [31]. Muscle masses and skin impedance are different among individuals. Therefore, the estimation model is required for each user.

#### 2.2.2. Estimation

In the estimation phase, the goal is to estimate the mean absolute values of target muscles. Users put on socks and set up the measurement system. Five channels of distal EMG signals ($a_{1}$, $a_{2}$, $a_{3}$, $a_{4}$, and $a_{5}$) are measured during tasks. $A_{i}$ is calculated, where *i* is the position label, from Equation (7). The band and moving widths are the same as those from the calibration phase. The mean absolute values $S(A_{i})$ and A array are calculated the same as they were in the calibration phase. The estimated signals $\hat{L}=\left[{\hat{l}}_{1},\;{\hat{l}}_{2}\;,{\hat{l}}_{3},\;{\hat{l}}_{4},\;{\hat{l}}_{5}\;,\;{\hat{l}}_{6}\right]$ are calculated using ***A*** and the estimation models $M_{L_{1}},\;M_{L_{2}},\;M_{L_{3}},\;M_{L_{4}},\;M_{L_{5}},\;M_{L_{6}}$ from the following equation.

(9) $$\hat{L}\left(k\right)={\left\{M_{L_{1}}\left[A\left(k\right)\right],\;M_{L_{2}}\left[A\left(k\right)\right],\;M_{L_{3}}\left[A\left(k\right)\right],\;M_{L_{4}}\left[A\left(k\right)\right],\;M_{L_{5}}\left[A\left(k\right)\right],\;M_{L_{6}}\left[A\left(k\right)\right]\right\}}^{T}$$

where *k* is the sampling index, and $\hat{L}\left(k\right)$ and A(k) are the *k*-th samples of $\hat{L}$ and ***A***.

## 3. Experiment

### 3.1. Electrode Size Test

Conductive-fabric electrodes have higher impedance than wet-type electrodes. Therefore, electrodes size is important for stable measurement. We conducted a pre-experiment for defining electrode size using one participant. Three sizes, 1 × 1 cm, 2 × 2 cm, and 3 × 3 cm, were tested. The participant wore a sock with conductive fabric electrodes attached on the right foot. Six sensors ($L_{1}$-$L_{6}$) were placed on muscle bellies, as shown in Figure 6. All electrodes were connected to a data logger. BioLog DL-2000 (S&ME Corp., Nakano, Japan) was used for data logging. The resolution was 16 bits. DL-2000 can measure signals from eight channels at a sampling rate of 1 [kHz]. Therefore, two DL-2000 data loggers were used. The EMG signals and distal EMG signals were measured in parallel when the participant conducted plantar flexion and dorsiflexion for 30 s for each electrode size. The signal processing was the same as in Section 2.1.1. The obtained ***L*** and ***A*** were split into two. One was used for model generation and the other for regression. We compared the correlation coefficients obtained using ***L*** and $\hat{L}$ for each electrode size.

### 3.2. Muscle Activity Estimation

As mentioned in Section 2.2, the mean absolute value of the target muscles is estimated based on distal EMG signals obtained using the proposed system. The accuracy of mean absolute value estimation is important for muscle activity analysis such as active phase analysis. Therefore, the estimation accuracy of the mean absolute value was evaluated. Electrodes might be displaced owing to body movements, and the displacement might affect the estimation accuracy. The effects of body movements on the estimation accuracy were also evaluated. In addition to the evaluations of signal estimation accuracy, the accuracy of spatio-temporal muscle activity analysis was evaluated.

There are six target muscles: tibialis anterior, gastrocnemius lateral muscle, gastrocnemius medial muscle, soleus lateral muscle, soleus medial muscle and peroneus muscle. Several walking situations (speed/load) occur according to the situations of people in daily life. In this experiment, four tasks were chosen as fundamental situations: “Walk (2 km/h)”, “Walk (3 km/h)”, “Walk (4 km/h)”, and “Walk (2 km/h) with load” for 60 s. In “Walk (2 km/h) with load”, participants carried a 7.5 kg bag on their shoulder. We obtained signals for four tasks from ten participants. Data loggers were attached on the subject’s waist, all electrodes were connected to the logger via wires. The electrodes placed on the target muscles were wrapped by tape. We recruited participants who had no problems walking; all participants were men with an average age of 25.0 years (std: 3.9), the average circumference of the ankle was 22.9 cm (std: 2.2 cm).

Ambu^®^Bluesensor Electrodes were used as wet-type electrodes, and they were placed at ***L*** positions, as shown in Figure 6. BioLog DL-2000 (S&ME Corp.) were used for measuring signals. The resolution was 16 bits. DL-2000 can measure signals from eight channels at a sampling rate of 1 [kHz]. We used sets of DL-2000 and one Windows PC to measure one side of the leg. The DL-2000 and PC were connected through USB. Both legs were measured at the same time. Each DL-2000 was synchronized based on digital triggers. After the wiring was complete, the lower leg was wrapped with a bandage to prevent movement artifacts. “Electric Walker AFW3309”, provided from ALINCO, was the treadmill used for the walking tasks. In this experiment, the estimation models $M_{L_{1}},\;M_{L_{2}},\;M_{L_{3}},\;M_{L_{4}},\;M_{L_{5}},and\;M_{L_{6}}$ satisfying Algorithm 1 were calculated using the library of “xgboost” [32]. Subjects wore the experimental instruments even between different tasks.

#### 3.2.1. Mean Absolute Values Estimation Accuracy

The purpose of this experiment was to evaluate the accuracy of muscle activity estimation based on mean absolute value using the proposed method. The distal EMG signals and EMG signals of the target muscles were measured at the same time for each task. The data of ***L*** and ***A*** were calculated using the measured signals. In this experiment, one estimation model was made for one subject. To construct the estimation model for one target subject, training data was constructed by combining the data of the target subject and other subjects. Each task data of the target subject was divided into two. One was used as training data and the other was used as test data. All data of other subjects was used for training. For data dependency, the same calculation was performed by replacing the training data and the test data of the target subject. Each of the two estimated data in each task for each subject were concatenated. The root mean squared error (RMSE) was used for evaluating the accuracy of each muscle as shown below.

(10) $$RMSE=\sqrt{\frac{1}{N}\sum_{i=1}^{N}{\left(y\left(i\right)-\hat{y}\left(i\right)\right)}^{2}}$$

where *N* is the length of data, y(i) is the *i*-th sample of the measured data, and $\hat{y}\left(i\right)$ is the *i*-th sample of the estimated data. To compare the proposed method, estimation based on the features extracted from a simple bandpass filter with passing frequencies from 20 to 500 Hz was used as a baseline.

#### 3.2.2. Dependability

The purpose of this experiment was to evaluate the effects of body movements on the estimation accuracy. In order to evaluate the impacts of body movements, the data of each task of each subject was split into two. The first half of the split data was used as the training data with low impacts of body movements compared to the second half of the data. The second half of the split data was used as the training data with high impacts of body movements compared to the first half of the data. In each task of each subject, two models were trained by using the first half and second half of the data of the task of the subject. All data of other subjects were combined with each split data of target subject. We calculated the accuracies of the two models in each task of each subject. Root mean squared errors were calculated from the estimated two data individually.

#### 3.2.3. Spatial and Temporal Analysis

**Figure 8:**
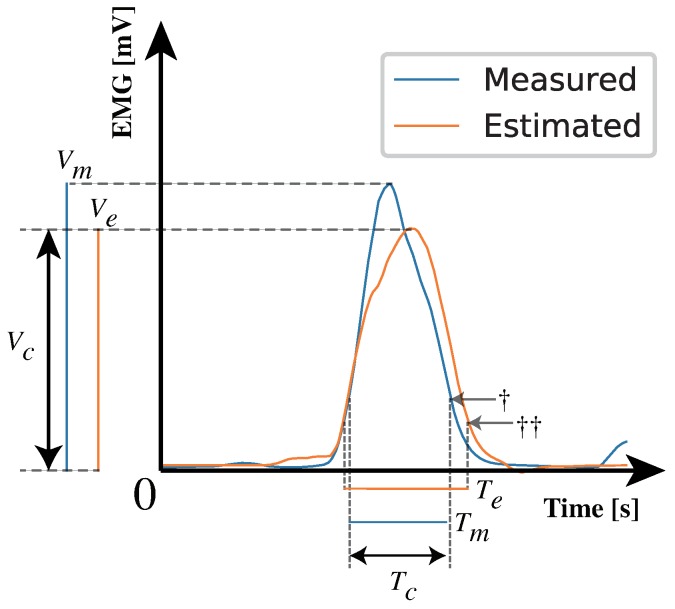
$T_{m}$ and $T_{e}$ are the times at which EMG values were measured and estimated over the thresholds. $V_{m}$ and $V_{e}$ are the maximum values for the measured and estimated EMG values over the thresholds, respectively. $T_{c}$ and $V_{c}$ are the time length and volume, respectively, where $T_{m}$ and $T_{e}$ and $V_{m}$ and $V_{e}$ overlap. †: A threshold for the measured EMG, ††: A threshold for the estimated EMG. Both thresholds are calculated based on a specific percentage.

A threshold-based muscle activation analysis is a simple approach to analyze muscle activity [33,34,35]. In this paper, we adopted a threshold-based temporal-spatial muscle activation analysis, as shown in Figure 8. A specific percentage of the amplitude of the signal was set as a threshold value. The specific percentage was defined as 20% arbitrarily. The time lengths exceeding the thresholds were calculated as $T_{m},T_{e}$ from the measured and estimated EMG values, respectively. Maximum EMG values exceeding the thresholds were calculated as $V_{m},V_{e}$ from the measured and estimated EMG. $T_{c},V_{c}$ are the time and volume, respectively, where $T_{m},T_{e}$ and $V_{m},V_{e}$ overlap. Temporal accuracy and spatial accuracy were calculated by

(11) $$Temporal\;accuracy=\frac{T_{c}}{T_{m}+T_{e}-T_{c}}$$

(12) $$Spatial\;accuracy=\frac{V_{c}}{V_{m}+V_{e}-V_{c}}$$

If there are multiple sections that exceed the threshold values, $T_{m},T_{e},V_{m},V_{e},T_{c}$ and $V_{c}$ are calculated in each section and their total values are used for the accuracy calculation. This analysis was conducted by using the estimated data obtained in Section 3.2.1. Temporal and spatial analyses are based on the detection of muscle activities. According to Godho et al., if the percentage decreases, the true positive rate of the muscle activity increases and the true negative rate of muscle activity decreases. In addition, if the percentage increases, the true positive rate of muscle activity decreases and the true negative rate of muscle activity increases [36]. It is supposed that lower percentages make the results better if analysis data contains many positives (muscle activities) and a few negatives (noises and estimation errors and so on). It is also supposed that higher percentages make the results better if analysis data contains a few positives and many negatives.

This experiment described in this section was conducted with approval from the ethics committee of the University of Tsukuba (2017R157) and NTT Service Evolution Laboratories (ERP-Ethics-17-013).

## 4. Results

### 4.1. Electrode Size Test

Table 2 shows the correlation coefficients for each electrode size. All *p*-values were less than 0.05. The 3 × 3 cm electrode size had the most stability compared to the other two sizes. Therefore, we adopted 3 × 3 cm as the electrode size.

### 4.2. Muscle Activity Estimation

#### 4.2.1. Mean Absolute Value Estimation Accuracy

The signals for only eight of the participants were used for analysis because the signals for the other two participants were noisy. Root mean squared error was calculated as Equation (10). The root mean squared errors of all tasks and all subjects were combined. Table 3 shows the median values of the root mean squared errors [mV] of each muscle in the proposed and baseline method. All root mean squared errors of the proposed method were significantly different from those of baseline because all *p*-values of Mann-Whitney U test for all muscles were less than 0.01.

Table 4 shows the median root mean squared errors [mV] of each task based on the proposed method. All errors were less than those of baseline. Each error was compared with that of baseline based on the Mann-Whitney U test. There were significant differences at several muscles in all tasks between the error of the proposed method and that of the baseline.

**Figure 9:**
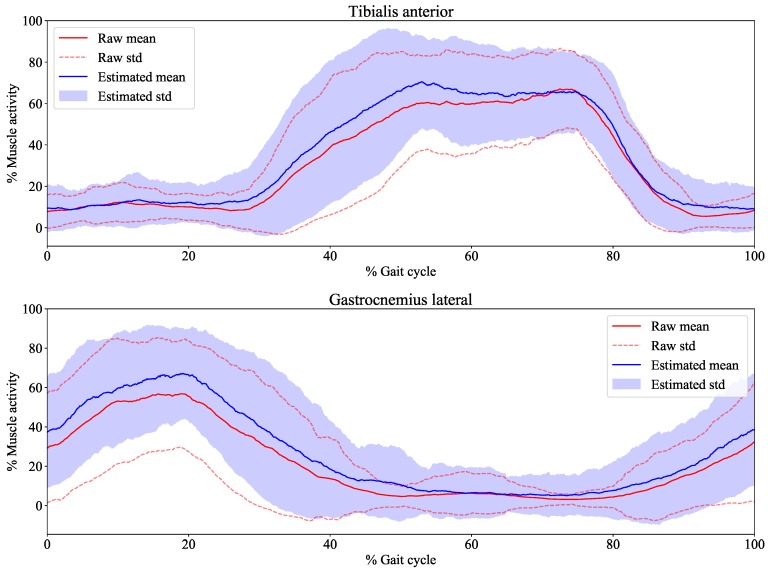
Average mean absolute values for the “Walk(2 km/h) with load” task. The red line is the average mean absolute value, the red dotted line is the standard deviation of mean absolute value, the blue line is the average regression value, and the purple area is the standard deviation of average regression value.

Figure 9 shows the trajectories of the average measured signal and average estimated signal under “Walk(2 km/h) with load” condition. Each muscle activity has a cyclic function during gait. Five cycles were extracted from all subjects based on a certain threshold, which was experimentally defined. All cycle data were normalized based on spatial and temporal aspects. Spatial normalization was calculated as the following equation.

(13) $$x^{\prime }=\frac{x-x_{min}}{x_{max}-x_{min}}\times 100$$

where $x^{\prime }$ is the normalized data, ***x*** is one cycle data extracted from the data of a subject’s muscle, $x_{max}$ is the maximum value of ***x***, $x_{min}$ is the minimum value of ***x***. Temporal normalization is performed so that the time of all data is 0–100, and liner interpolation is performed so that the sampling rate is 1000 [Hz]. The average measured signal and average estimated signal were calculated from the normalized data of all subjects. As shown in Figure 9, the trend of the average estimated signals was close to that of average measured signals.

#### 4.2.2. Dependability

Root mean squared errors were calculated from each task of each subject. Table 5 shows the median root mean squared errors of each muscle of the first model and the second model. There were a few differences between the first model accuracy and the second model accuracy. In addition, there were no significant differences because all *p*-values of the Mann-Whitney U test between the accuracy of the first model and the second model were greater than 0.05.

#### 4.2.3. Spatial and Temporal Analysis

Table 6 and Table 7 shows the median temporal accuracy and spatial accuracy from all subjects by using the estimated signals based on the proposed method. All accuracies were higher than those of the baseline. Both from the temporal and spatial point of view, most results were higher than 80%. There were significant differences at several muscles in all tasks between the accuracy of the proposed method and that of the baseline.

## 5. Discussion

The goal of this study was to develop a sock-type system for estimating lower leg muscle activity based on distal EMG signals around the ankles. Multiple band-pass filter-based feature extraction was proposed for increasing the estimation accuracy. We conducted experiments involving five channels with conductive fabric for obtaining distal EMG signals, and we estimated the mean absolute value signals of target muscles (tibialis anterior, gastrocnemius lateral, gastrocnemius medial, soleus lateral, soleus medial, and peroneus ). In the experiment of the mean absolute value estimation accuracy, the estimation accuracy of the proposed method was compared with that of a baseline method that is based on a single bandpass filter. As shown in Table 3 and Table 4, the accuracies of the proposed method were higher than those of the baseline method. This result indicates that the proposed method can estimate mean absolute values at a high accuracy than the baseline method. In addition, this result indicates that the proposed method is applicable to all conducted tasks in this experiment at high accuracy. As shown in Figure 9, the trend of the estimated signals from our system is close to that of the measured signals. The variance of the average measured signal and the average estimated signal are large, as shown in Figure 9. We considered that this was caused from the individual variability of muscle activity in the gait. This result indicates that our system is user-dependent owing to the individual differences of muscle activity in the gait.

In the dependability analysis, the effect of body movements on estimation accuracy were evaluated by constructing two estimation models. As shown in Table 5, there were no significant differences between the first model (assumed to be low impacts of body movements) and the second model (assumed to be high impacts of body movements). Therefore, the effects of body movement on the estimation accuracy can be considered to be small. This result is assumed to depend on the kinds of tasks, the body characteristics of subjects, the duration of tasks, and the size of socks. When the above conditions are determined, the dependability test is carried out. Most results from the temporal-spatial muscle activity analysis exceeded 80%, as shown in Table 6 and Table 7. Therefore, our system can be used to analyze information such as muscle-force ratio, muscle activation (ON/OFF) state, and the timing of muscle contraction and relaxation. In this experiment, the same tasks were used for calibration and estimation. Our system estimates EMG signals of target muscles based on distal EMG signals, which are biosignals propagated from several muscles. Therefore, distal EMG signals are defined by the contraction of each muscle. The results indicate that target muscle activity under the same muscle-contraction patterns as calibration can be estimated with high accuracy. Movement composed of the same muscle-contraction patterns as those of the target movement is assumed to be an appropriate calibration movement.

We applied multiple band-pass filters to the distal EMG signals. Since good results were obtained using our system, separating raw distal EMG signals into multiple frequency bands is considered beneficial. Raw distal EMG signals are assumed to contain several signals whose frequency distributions differ. One reason for this is that noise of a specific frequency distribution has been separated. Another is that the frequency distributions of each target muscle might originally be different. A third reason is that frequency distributions slightly change over the course of signal propagation, even if the frequency distributions of the original signals are the same. It is said that the frequency distributions of EMG signals are affected by propagation velocity [37]. It is assumed that longer the propagation path of the EMG signals, the more susceptible they are to the effect of propagation velocity. Although computational cost is considered to be a concern, it is not supposed to be a major problem in the offline analysis as in this experiment.

The reason the data of the two subjects were noisy is attributed to the fact that the socks did not fit them. Table 8 summarizes the length around the ankle [cm] for each subject. Two subjects whose data were noisy were subjects B and D, and their lengths around the ankle were shorter than others. The necessity to choose suitable socks could be a limitation of the proposed system. The impact of sweat also could be a limitation. It is assumed that the effect of sweat on these electrodes may cause an increase in the biopotential voltage measured from the surface of the skin with a decrease in skin impedance. The correction of biopotential voltage based on impedance analysis is considered to reduce the impact of sweat. The necessity of the calibration and recalibration is supposed to depend on whether the same signal characteristics as in the calibration are measured. Biopotential signals, which are measured from the surface of the skin, vary depending on muscle masses, skin impedance, and the coordination of muscle activity. It is assumed that they are different among individuals, and therefore, the estimation model should be calibrated for each user. There is also a possibility that the shifts or rotations of electrodes by re-wearing the socks. The impact of the shifts or rotations of electrodes should be investigated in further study.

## 6. Conclusions

The aim of this research was to develop a sock-type system for estimating lower leg muscle activity based on distal EMG signals around the ankles. A sock-type wearable sensor with electrodes implemented around the ankle was proposed as an easy-to-wear system. By placing electrodes only around the ankle, a less restrained wearable system was realized. Owing to electrode position limitations, distal EMG signals around the ankle were measured while EMG signals on each muscle belly could not be measured. We constructed an estimation system for lower leg muscle activity based on distal EMG signals. Multiple band-pass filters were adopted for increasing the estimation accuracy of the proposed system. The estimation accuracy of the mean absolute values, dependability, and the temporal-spatial analysis of the proposed system were validated through the experiment. From the results, we could confirm that the proposed system can be used for estimating muscle activity at a high accuracy.

It can be a disadvantage in that the system requires a large high-compression area owing to the distribution of the target muscles. A distal EMG signal analysis allows for the redesign of electrode positions based on the propagation characteristics of EMG signals. Therefore, distal EMG signals-based analysis has the potential to accelerate the popularization of wearable muscle activity monitoring systems.

## Figures and Tables

**Table 1:** Relationships between sensor labels and muscles.

Label	Muscle
$L_{1}$	Tibialis anterior
$L_{2}$	Gastrocnemius lateral muscle
$L_{3}$	Gastrocnemius medial muscle
$L_{4}$	Soleus lateral muscle
$L_{5}$	Soleus medial muscle
$L_{6}$	Peroneus muscle

**Table 2:** Correlation coefficients under electrode size conditions.

**Size**	**Tibialis Anterior**	**Peroneus**	**Gastrocnemius Lateral**
1 × 1 cm	0.449	0.740	0.777
2 × 2 cm	0.932	0.834	0.943
3 × 3 cm	0.946	0.889	0.950
**Size**	**Gastrocnemius Medial**	**Soleus Lateral**	**Soleus Medial**
1 × 1 cm	0.755	0.736	0.756
2 × 2 cm	0.916	0.930	0.901
3 × 3 cm	0.952	0.962	0.956

**Table 3:** Median root mean squared errors [mV] of the proposed and baseline method (** *p* < 0.01).

	**Tibialis Anterior**	**Gastrocnemius Lateral**	**Gastrocnemius Medial**
Proposed	0.0125 **	0.0186 **	0.0349 **
Baseline	0.0167	0.0220	0.0498
	**Peroneus**	**Soleus Lateral**	**Soleus Medial**
Proposed	0.0130 **	0.0102 **	0.0298 **
Baseline	0.0170	0.0141	0.0459

**Table 4:** Median root mean squared errors [mV] of each task based on the proposed method (* *p* < 0.05, ** *p* < 0.01).

	**Tibialis Anterior**	**Gastrocnemius Lateral**	**Gastrocnemius Medial**
2 km	0.0121 **	0.0151	0.0372 *
3 km	0.0131 *	0.0184	0.0369 *
4 km	0.0176	0.0237	0.0338
load	0.0110 **	0.0175	0.0338
	**Peroneus**	**Soleus Lateral**	**Soleus Medial**
2 km	0.0139	0.0095 **	0.0269 *
3 km	0.0113 *	0.0107 **	0.0300 *
4 km	0.0176	0.0127 **	0.0347 *
load	0.0112	0.0084 **	0.0255

**Table 5:** Median root mean squared errors of the first half and the second half.

	**Tibialis Anterior**	**Gastrocnemius Lateral**	**Gastrocnemius Medial**
First model	0.0111	0.0175	0.0351
Second model	0.0107	0.0164	0.0331
	**Peroneus**	**Soleus Lateral**	**Soleus Medial**
First model	0.0100	0.0076	0.0269
Second model	0.0100	0.0088	0.0251

**Table 6:** Median temporal accuracy [%] of each task of the proposed method (* *p* < 0.05, ** *p* < 0.01).

	**Tibialis Anterior**	**Gastrocnemius Lateral**	**Gastrocnemius Medial**
2 km	0.896 *	0.762	0.773
3 km	0.923 **	0.824	0.843
4 km	0.937 *	0.852	0.837 *
load	0.911 **	0.788	0.792 *
	**Peroneus**	**Soleus Lateral**	**Soleus Medial**
2 km	0.820	0.931	0.861
3 km	0.861 *	0.939 *	0.885 *
4 km	0.823	0.910 *	0.896
load	0.796	0.933	0.858

**Table 7:** Median spatial accuracy [%] of each task of the proposed method (* *p* < 0.05, ** *p* < 0.01).

	**Tibialis Anterior**	**Gastrocnemius Lateral**	**Gastrocnemius Medial**
2 km	0.866 *	0.779	0.790 *
3 km	0.877 *	0.818	0.819 **
4 km	0.860	0.794	0.835
load	0.880 *	0.773	0.765 **
	**Peroneus**	**Soleus Lateral**	**Soleus Medial**
2 km	0.844	0.887 *	0.830 *
3 km	0.858	0.911 *	0.858 *
4 km	0.855	0.901	0.842 *
load	0.835 *	0.899 *	0.827 *

**Table 8:** Length around the ankle of each subject.

Subject	A	B	C	D	E	F	G	H	I	J
Length around the ankle [cm]	24.7	19.5	26.0	19.5	21.9	25.3	23.8	24.2	21.5	23.0
